# Quarterly Report of Proceedings

**Published:** 1856-10

**Authors:** 


### QUARTERLY REPORT OF THE PROCEEDINGS.

### PAPERS READ AT THE ORDINARY MEETINGS.

*Monday, July 7th,* 1856. "On the Principles of Inductive Philosophy as applied to the Study of Epidemics." By B. W. Richardson, M.D.

*Monday, August 4th,* 1856. "Report on Cholera which visited Her Majesty's Black Sea Fleet in the Autumn of 1854; compiled from the Returns of the Medical Officers of the Fleet to Queries drawn up by the Cholera Committee of the Epidemiological Society, and sent out by order of Government." By B. G. Babington, M.D.

In the discussion which followed the President's paper, Dr. Milroy observed on the remarkable immunity of the officers, as compared with the men, from attacks of cholera in those vessels of the Black Sea fleet which had suffered the most. He was not surprised at this himself, and in his opinion the cause was sufficiently obvious, viz., the much larger amount of breathing space in the quarters of the former, more especially at night, when the necessity for pure air is even greater than during the day. He had carefully gone over the *Royal Albert* and the *Queen,* the admirals' ships at the Kameisch and the Bosphorus, in company with the medical officers, in the latter case with Dr. Deas, the Inspector-General of the fleet, whose views entirely concurred with his own. Take the case of a three-decker with 1,000 or 1,200 men on board. More than two-thirds of them are stowed away on the lower deck, which is necessarily the worst ventilated, whilst the rest, consisting chiefly of the marines and boys, are berthed in part of the middle deck. The whole of the upper deck and one-half of the middle deck are left quite free and unoccupied. There was no good reason why so much space should not be made available for the comfort and the health of the men at night, and he trusted that ere long it would be made use of at all times. He had spoken with the medical officers, and with the captains and lieutenants on this subject, and he had found that it was only because it had been always the practice in the service, that no change was made, for all admitted that the atmosphere in the between decks, after the men had turned into their hammocks, was most disgusting. Such being the case, could we wonder that diseases like cholera and yellow fever sometimes prevailed to a frightful extent in vessels of war?

The history of the terrible outbreak in the *Britannia* at Varna, in 1854, was a melancholy example. All the ports of the lower deck had been shut, in consequence of the weather becoming stormy, and the result was, that the air which the men breathed was positively pestiferous. The ventilation of the between decks in all ships might be much improved, and by very simple means, when the ship was being built; but, unfortunately, little or no attention was paid to this point by shipwrights, and everything was sacrificed, either to mere appearance, or the working of the guns, or the stowing away of the stores or cargo. In closing his remarks he would offer one practical hint, which he believed to be of great importance, viz., that whenever a ship of war happened to be in, or to arrive at a place where any epidemic disease existed, the crew should immediately be spread over all the available space in the different decks. In some cases, as when the vessel is lying in port, it may even be advisable to have some of the crew to sleep on the spar deck, under an awning; disease would then be often prevented altogether, and at all events be very materially diminished. No other sanitary appliance could be of much avail if the free and abundant supply of fresh air to the men at night was not attended to.

---

### PRESENTATIONS.

"Memoir on the Cholera at Oxford in the year 1854", by H. W. Acland, M.D. Presented by the author.

"India as it ought to be", by Major William Hough. Presented by the author.

---

### NEW MEMBERS.

The following gentlemen have been elected members of the Society during the quarter:—

*Resident Members.*—J. W. Tripe, M.D., King's Cross, Commercial Road; C. J. B. Aldis, M.D., 1, Chester Terrace, Chester Square; Thomas Ansell, M.D., Harley Place, Bow; John Challice, M.D., Bermondsey; Henry Northover Pink, Esq., M.R.C.S., Greenwich.

*Corresponding Members.*—A. Von Iffiand, M.D., Beaufort, New Quebec; W. Isidore Cox, Esq., M.R.C.S., Hindley, near Wigan, Lancashire.

